# A whole cell-based Matrix-assisted laser desorption/ionization mass spectrometry lipidomic assay for the discovery of compounds that target lipid a modifications

**DOI:** 10.3389/fmicb.2023.1156795

**Published:** 2023-04-17

**Authors:** Wenhao Tang, Joanne Osborne, Laurent Dortet, Gerald Larrouy-Maumus

**Affiliations:** ^1^Faculty of Natural Sciences, Department of Mathematics, Imperial College London, London, United Kingdom; ^2^Life Arc, London, United Kingdom; ^3^Department of Bacteriology-Hygiene, Bicêtre Hospital, Assistance Publique–Hôpitaux de Paris, Le Kremlin-Bicêtre, France; ^4^Faculty of Natural Sciences, Department of Life Sciences, Centre for Bacterial Resistance Biology, Imperial College London, London, United Kingdom

**Keywords:** polymyxin, MALDI, lipid A, inhibitors, lipidomics, *E. coli*, whole-cell assay

## Abstract

**Introduction:**

Matrix-assisted laser desorption/ionization-time of flight mass spectrometry (MALDI-TOF MS) is a powerful analytical technique that has been applied to a wide variety of applications ranging from proteomics to clinical diagnostics. One such application is its use as a tool for discovery assays, such as monitoring the inhibition of purified proteins. With the global threat from antimicrobial-resistant (AMR) bacteria, new and innovative solutions are required to identify new molecules that could revert bacterial resistance and/or target virulence factors. Here, we used a whole cell-based MALDI-TOF lipidomic assay using a routine MALDI Biotyper Sirius system operating in linear negative ion mode combined with the MBT Lipid Xtract kit to discover molecules targeting bacteria that are resistant to polymyxins, which are considered last-resort antibiotics.

**Methods:**

A library of 1200 natural compounds was tested against an *E. coli* strain expressing *mcr-1*, which is known to modify lipid A by adding phosphoethanolamine (pETN), making the strain resistant to colistin.

**Results and Discussion:**

Using this approach, we identified 8 compounds that led to a decrease in this lipid A modification by MCR-1 and could potentially be employed to revert resistance. Taken together, as-proof-of-principle, the data we report here represent a new workflow based on the analysis of bacterial lipid A by routine MALDI-TOF for the discovery of inhibitors that could target bacterial viability and/or virulence.

## Introduction

Antibiotic resistance is an issue of global importance and one of the defining public health concerns of our time (Rochford et al., 2018). The limited pipeline of novel antimicrobials and the spread of multidrug-resistant (MDR) organisms have increased our reliance on a few last-resort antibiotics for the treatment of MDR gram-negative bacteria. The principal last-resort agents are the polymyxin antibiotics polymyxin B and colistin (Falagas and Kasiakou, 2005; Kontopidou et al., 2014).

In gram-negative bacteria such as *Escherichia coli*, polymyxin resistance mostly occurs as a consequence of lipopolysaccharide (LPS) modifications in the form of the addition of the cationic groups phosphoethanolamine (pETN) and/or 4-amino-L-arabinose (L-Ara4N) to the lipid A portion of LPS (Olaitan et al., 2014; Jeannot et al., 2017). These lipid A modifications often arise due to alterations to the PmrAB and PhoPQ two-component systems, mutations to the negative regulator of PhoPQ, MgrB, or due to the activity of plasmid-borne pETN transferases called mobile colistin resistance (MCR) enzymes (Poirel et al., 2017). The first MCR enzyme, MCR-1, was reported in 2016 (Liu et al., 2016), and this discovery was followed by the rapid identification of other mobile polymyxin resistance genes (Yang et al., 2018; Carroll et al., 2019; Zhang et al., 2019a,b), which pose serious challenges and cause pressure in health care systems.

The increasing number of polymyxin-resistant bacteria and the loss of last line-of-defense antibiotics require the development of innovative strategies to address the serious challenges posed by these bacteria. Targeting lipid A modifications with potent inhibitors will be a game changer in the race against superbugs. One such strategy is to directly inhibit MCR proteins using small molecules. For example, Zhou et al. (2019) reported the discovery of osthol as a new MCR-1 inhibitor that, when used in combination with polymyxins, is able to restore their ability to kill *mcr-1*-positive Enterobacterales. Similarly, based on the structural characterization of EptA, the lipooligosaccharide phosphoethanolamine transferase A which catalyzes the addition of pETN in *Neisseria* sp., Mullally et al. (2022) used a library of small-fragment compounds to test for their binding to EptA and their ability to enhance susceptibility of the reference strain *Neisseria gonorrhoeae* FA1090 to polymyxin B. They identified three compounds that enhanced susceptibility of the strain to polymyxin B by 4-fold including one compound that, when exposed to that compound, caused FA1090 to exhibits a 17% reduction of lipid A modified with pETN as measured by MALDI-TOF MS (Mullally et al., 2022).

Such an approach is of interest because it exploits the ability of a new inhibitor to decrease lipid A modification by pETN, enabling polymyxins to bind to lipid A, which leads to the killing of the pathogen.

We previously reported the development of the MALDIxin test, which uses matrix-assisted laser desorption/ionization-time-of-flight (MALDI-TOF) mass spectrometry to rapidly detect lipid A and its modifications (Dortet et al., 2018a,b,c, 2020a; Furniss et al., 2019; Jeannot et al., 2021). Here, we use this technology to discover new compounds that interfere with lipid A biosynthesis.

The aim of this study was to test the low-resolution linear mode employed by the MALDI Biotyper Sirius system (Bruker Daltonics) operating in negative ion mode combined with the commercial MBT lipid Xtract kit (Bruker Daltonics), which has potential as a tool for the discovery of new compounds that interfere with lipid A modifications directly or indirectly by monitoring these modifications in an *E. coli* strain expressing *mcr-1* using whole bacteria instead of purified enzymes.

## Materials and methods

### Bacterial strain and culture conditions

*Escherichia coli* J53 expressing the *mcr-1* gene was provided by the Centre National de Reference associé Résistance aux Antibiotiques “Entérobactéries productrices de carbapénèmases.” Bacteria were grown in tryptic soy broth (TSB) in a shaking incubator set to 37°C and 180 rpm.

### Compound library

The Phytoquest library^1^ was obtained from Life Arc (London, United Kingdom). The library was the Phytopure library from Phytoquest (see text footnote 1), which comprises 1,200 pure (purity >90%) and mainly non-polar compounds obtained from natural origins with structures that have been validated through 1H and 2D 1H-13C NMR spectroscopy dissolved in DMSO.

### Assay conditions and extraction of lipid A

A total of 195 μl of overnight bacterial culture grown in TSA and diluted 1/100 was loaded into a 96-well plate (Grenier BioOne). Five microliters of the compound stocks, equivalent to a final concentration of 250 μM for compounds ID_1 to ID_880 or 62.5 μM for compounds from plates ID_881 to ID_1,200, or 5 μl of DMSO as a control, was added to the bacterial solution. The plates were then placed into a plate reader set to 37°C, and the OD at 600 nm was measured every 30 min over a period of 180 min. Then, the plates were centrifuged for 10 min at 1,000 × g at 4°C, and the supernatant was discarded.

As a control, osthol (O9265, Sigma–Aldrich) was used at a final concentration of 64 μg/ml from an original 10 mg/ml stock in ethanol.

Lipid A from *mcr-1*-positive *E. coli* J53 *mcr-1* was extracted using a modified MBT Lipid Xtract™ kit (Part No. 1889112). the pellets were transferred to 100 μl of lysis buffer consisting of 1% acetic acid in water (v/v) and placed in a PCR heat block for 20 min at 98°C. Once hydrolysis was achieved, the plates were centrifuged for 10 min at 1,000 × g at 4°C, and the supernatant was discarded. The hydrolysates were then washed once with 100 μl of water and suspended in 20 μl of water.

Next, 0.4 μl of the bacterial suspension was loaded onto an MSP 96 polished steel target (Bruker Daltonics, Part-No. 8280800), and 0.8 μl of MBT Lipid Xtract™ matrix (Part No. 1889112) was added to the top of the bacterial suspension, which was finally dried under a gentle stream of air.

### Acquisition of MALDI-TOF mass spectra

The spectra were recorded in the linear negative ion mode (laser intensity 60%, ion source 1 = 15.00 kV) with the MALDI Biotyper® Sirius system. Each spectrum corresponded to ion accumulation from 200 to 1,000 laser shots randomly distributed on the spot.

For visual inspection, the spectra obtained were processed with default parameters using flexAnalysis v.3.4 software (Bruker Daltonics, Germany).

### Data analysis

MALDIquant (Gibb and Strimmer, 2012) was used to preprocess the data. Data were first sqrt-transformed *via* “transformIntensity”; intensities were then smoothed *via* the “smoothIntensity” function with a half window size set to 20. Baselines were removed with the function “removeBaseline” with the “SNIP” method; then, intensities were calibrated *via* the “calibrateIntensity” function. Finally, spectra were aligned with the “alignSpectra” function. The mean intensities within ±5 units of *m*/*z* 1796 and *m*/*z* 1919 were calculated, and the effect of each compound was determined as a ratio as depicted in Equation 1. The doubling time was determined using Equation 2.

Equation 1


$$Ratio=\frac{\;\left(\frac{\frac{m}{z}1919}{\frac{m}{z}1796}\right)treated}{\left(\frac{\frac{m}{z}1919}{\frac{m}{z}1796}\right)DMSO}$$

Equation 2


$$Doublingtime=30\times \left(\frac{\;\left(\log\;2\right)}{\left(\log\;(ODn+1\right)-\log\left(ODn\right))}\right)$$

## Results and discussion

### Osthol decreases lipid a modification by MCR-1

**Figure 1:**
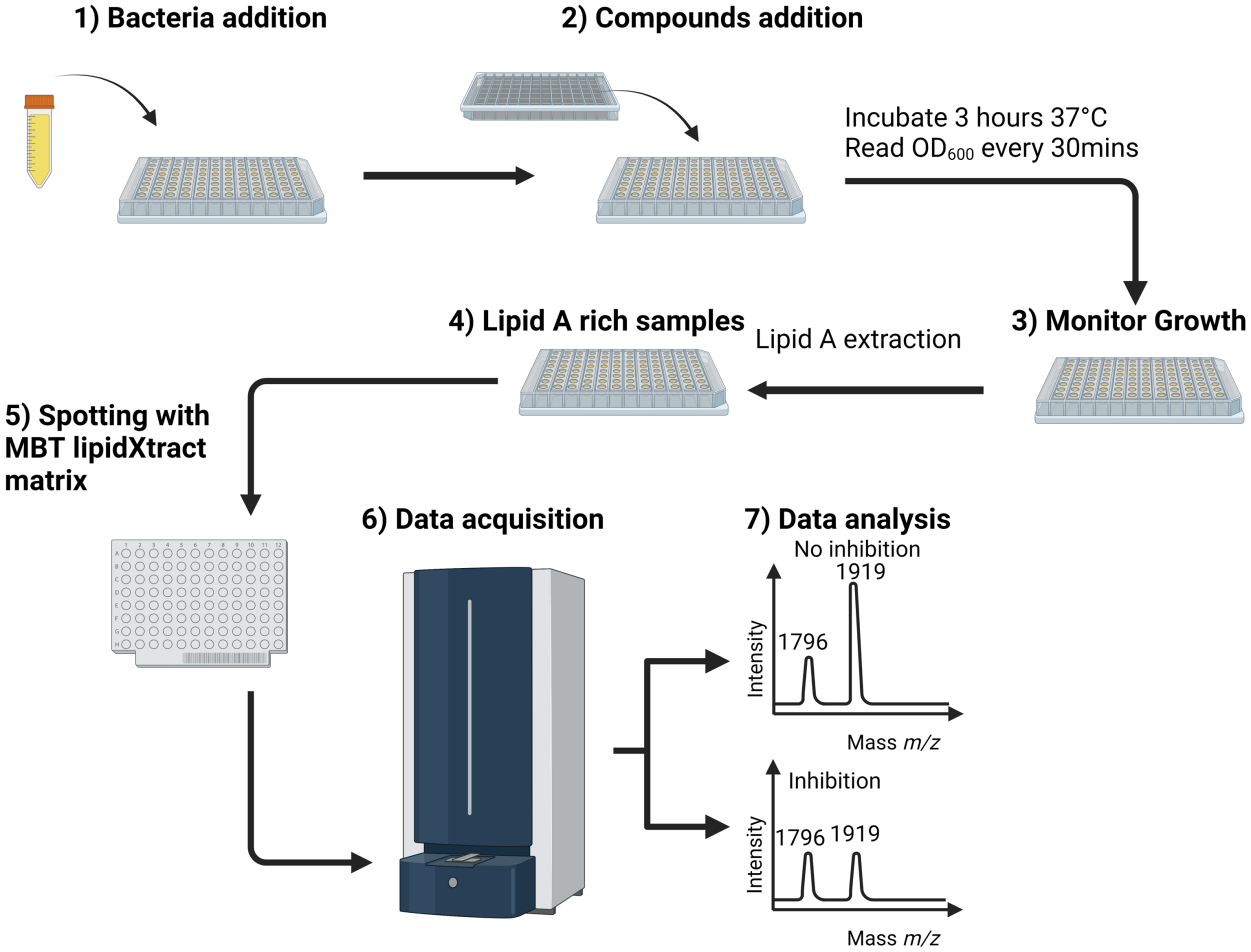
MALDI-TOF MS-based lipidomics screening workflow for whole-cell bacteria assays. After bacterial addition (overnight culture diluted 1/100) (1), the natural compounds were added at a predetermined final concentration (2), and the suspension was incubated for 3 h at 37°C in a spectrometer to monitor the optical density at 600 nm every 30 min (3). Once the measurements were complete, the bacterial suspensions were submitted to lipid A extraction (Jeannot et al., 2017), and the results of that process were spotted onto a MALDI target plate with MBT Lipid Xtract matrix (5). The MALDI target plate was introduced into the MALDI Biotyper Sirius system, and the samples were analyzed in linear negative ion mode (6). In the mass spectrum, active and inactive compounds can be distinguished based on the observed value of the ratio. Using this workflow, the total time to screen a plate containing 96 compounds was ~4 h, of which 3 h was needed for growth of the bacteria in presence of the compound of interests and ~ 1 h to process and acquire the samples for lipid A analysis by MALDI-ToF MS. Calculated by Equation 1 (7).

**Figure 2:**
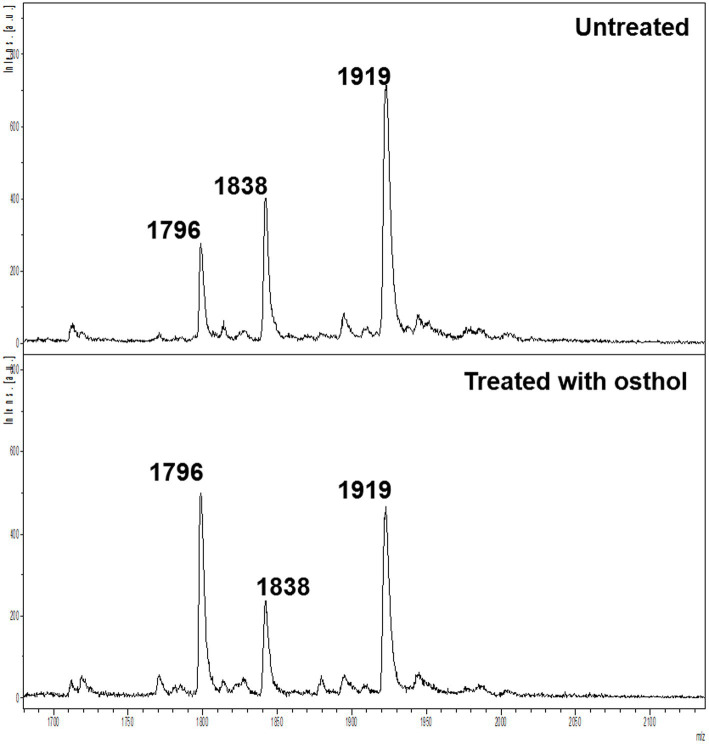
Mass spectra of untreated *E. coli* J53 *mcr-1* (top) and *E. coli* J53 *mcr-1* treated with 64 μg/ml osthol (bottom).

We used osthol, a known MCR-1 inhibitor, as a proof-of-principle to test whether monitoring the proportion of lipid A modified by MCR-1 can be used as a readout for the discovery of new molecules that affect lipid A modification. The workflow (Figure 1) was used to generate the mass spectra processed using Equation 1. A ratio of 1 indicates that the compound has no effect on the modification of lipid A. However, if this ratio is lower than 1, we can conclude that the compound has some potency against MCR-1 producers. As expected, in the case of osthol, we observed a decrease in the peak at *m*/*z* 1919.2 assigned to hexa-acyl diphosphoryl lipid A containing four C14:0 3-OH, one C14:0, one C12:0, and one residue of pETN (+123 mass units) from the unmodified *m*/*z* 1796.2 consisting of hexa-acyl diphosphoryl lipid A containing four C14:0 3-OH, one C14:0, and one C12:0. When exposed to osthol and compared to untreated samples, the calculated ratio was 0.56 ± 0.18 (Figure 2), suggesting that our approach can be used to screen for new compounds that interfere with pETN-mediated lipid A modifications.

### Growth assay combined with MALDI lipidomics identified natural compounds that affect bacterial growth and decrease the proportion of lipid a with pETN modifications

**Figure 3:**
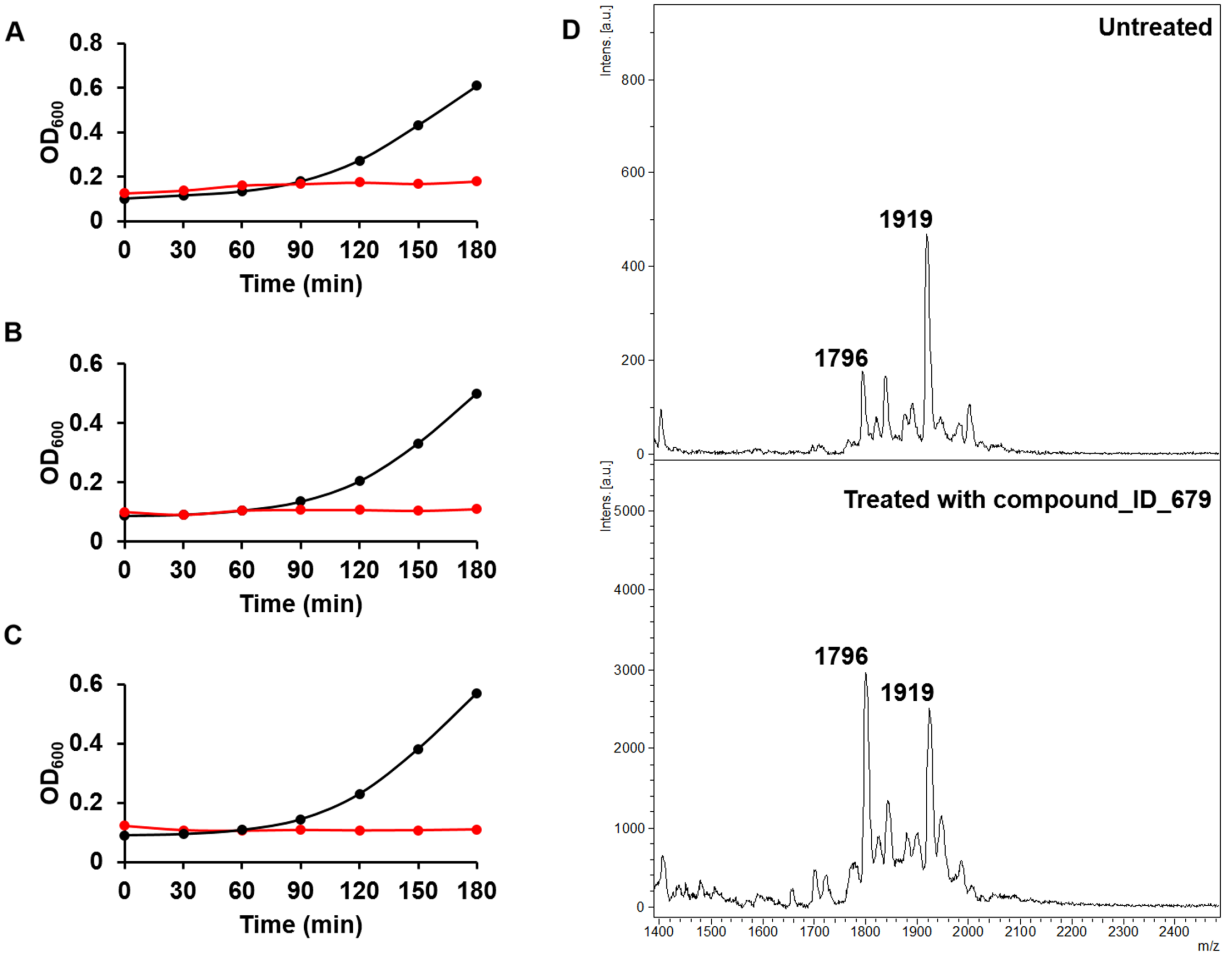
Phenotypic assays for the discovery of new compounds targeting mcr-1-producing *E. coli.* **(A)** Growth curves of untreated (black) and compound ID_100-treated *E. coli* (red). **(B)** Growth curves of untreated (black) and compound ID_264-treated *E. coli* (red). **(C)** Growth curves of untreated (black) and compound ID_499-treated *E. coli* (red). **(D)** Mass spectra of untreated (top) and compound ID_679 (250 μM)-treated *E. coli* J53-mcr1 (bottom).

Having demonstrated using osthol that the ratio proposed in Equation 1 can be used to discover molecules that could interfere with lipid A modification, we tested our workflow using the Phytoquest library (see text footnote 1), which contains 1,200 compounds of natural origin. We monitored bacterial growth in the presence of the compounds and calculated the ratio presented in Equation 1 as a readout of bacterial viability and direct or indirect interference with lipid A modification. By using this approach, out of the 1,200 screened compounds, three compounds, namely ID_100, ID_264, and ID_499, abolished the growth of *mcr-1*-expressing *E. coli* J53 at 250 μM (Figures 3A–C). The three compounds that lead to a growth defect could possibly target similar essential cellular processes such as replication, transcription, translation, or cell envelope biosynthesis. However, more work has to be done in order to underpin the detailed mode of action of those natural compounds. More interestingly, although no drastic changes in doubling time were noticed for the other molecules tested (Supplementary Table S1) with a threshold ratio set at 0.3 as a stringent parameter, eight compounds were found to drastically decrease the proportion of lipid A containing pETN (Table 1). For example, in the presence of compound ID_679 at a concentration set at 250 μM and compared to untreated bacteria, a large decrease in the peak at *m*/*z* 1919 was observed (Figure 3D) with a ratio of 0.27. It is important to stress that due the absorbance at OD 600 nm of a few of the compounds tested, the calculation of the doubling was not possible (Supplementary Table S1). In those cases, we therefore recommend the plate for CFU/mL to have the precise number of viable cells. When *E. coli* J53 *mcr-1* was treated with either compound ID_100 and ID_499, the doubling time increased and led to a lower ratio of lipid A modified by pETN. That could be caused by a change in metabolism or gene expressions of *E. coli* upon exposure to those molecules leading to direct or indirect changes in the fraction of lipid A pETN; more investigations should be done in order to determine the mode of action of those two natural compounds.

**Table 1:** Compounds that exhibited the largest decrease in the ratio of lipid A modified by MRC-1.

Compound ID	Ratio
667	0.22
427	0.23
340	0.25
284	0.27
689	0.27
679	0.27
659	0.27
347	0.29

This study demonstrates the versatility of the routine MALDI mass spectrometry Biotyper Sirius system in negative ion mode combined with the MBT Lipid Xtract kit for the discovery of new molecules that could interfere with lipid A modification. However, this study has some limitations. It remains to be determined whether the molecules identified inhibit MCR-1 directly or alter the regulation of lipid A biosynthesis. Their potency in combination with polymyxins must also be tested, as previously described (Zhou et al., 2019). The natural compounds were tested against replicating bacteria and, in subsequent studies, hit compounds should be tested against lag, logarithmic, and stationary phases of bacterial growth. Here, we tested *E. coli* containing the *mcr-1* gene, and it would be of interest to test the new molecules on bacteria expressing other *mcr* variants and other gram-negative bacterial species that have been reported to encode such *mcr* variants (e.g., *Salmonella enterica* or *Klebsiella pneumoniae*) or other *mcr*-like genes (e.g., overexpressed *eptA* in *Acinetobacter baumannii*; Dortet et al., 2018a, 2020a,b). Indeed, since the discovery of *mcr-1* in 2016 (Liu et al., 2016), ten *mcr* variants (*mcr-1* to *mcr-10*) have been reported to date (Hussein et al., 2021; Martiny et al., 2022; Rhouma et al., 2023). The compounds identified here should also be tested against chromosomally encoded resistance mechanisms (e.g., addition of L-Ara4N) and with a larger panel of relevant clinical isolates. In addition, the effect on bacterial growth and on lipid A modifications could be uncoupled. Here, in this study, we wanted to provide a new workflow to identify compounds that target *E. coli* mcr-1 by either inhibiting the growth of the bacteria or inhibiting the MCR-1 protein. That is why the compounds from the library used in this study should also be tested against strains that are susceptible to polymyxins to determine their effect on bacteria growth. The matrix used in this study is optimized for the analysis of bacterial lipid A in the negative ion mode. We cannot rule out that, as MALDI is semi-quantitative and validation of the hits should be validated using conventional methods (Hankins et al., 2013; Novikov et al., 2017; Powers et al., 2019).

This study expands the use of MALDI mass spectrometry for the discovery of inhibitors. For example, MALDI-TOF has been successfully applied to the discovery of inhibitors of the innate immune response mediator GMP-AMP synthase (cGAS), of which its mutation/dysregulation has been causally correlated to several inflammatory disorders. cGAS inhibition was evaluated by direct quantification of the physiological reaction product cyclic GMP-ATP (cGAMP; Simon et al., 2020). A similar approach has been used by Muller and colleagues for the discovery of inhibitors of the antigen aminopeptidase ERAP1, an important target in immuno-oncology and autoimmune diseases, by measuring inhibition of the enzyme of interest by MALDI-TOF (Muller et al., 2022). More recently, MALDI-TOF has been used to identify potent and selective modulators of deubiquitylating enzymes that play a vital role in the ubiquitin pathway by editing or removing ubiquitin from their substrate (De Cesare et al., 2020; De Cesare and Davies, 2023). One major limitation of these assays is their use of purified enzymes, which does not take into consideration drug penetration into biological systems such as cells or bacteria. To overcome this major hurdle, as proof-of-principle, we proposed a whole cell-based assay that directly measures the ratio of modified to unmodified lipid A in whole microorganisms compared to untreated controls. Nevertheless, once identified using a whole cell-based assay, *in vitro* enzymatic assays must be performed to confirm the interactions and evaluate the off-target effects.

To conclude, by using a whole-cell-based MALDI lipidomics approach, we proposed a new and innovative alternative for the discovery of new inhibitors of lipid A and LPS biosynthesis in gram-negative bacteria that can target cell viability and/or the virulence and resistance of pathogens.

## Data availability statement

The original contributions presented in the study are included in the article/Supplementary material, further inquiries can be directed to the corresponding author.

## Author contributions

GL-M designed and performed the experiments. JO provided the phytoquest compound library (www.phytoquest.co.uk). LD provided the *Escherichia coli* strain. WT and GL-M analyzed the data. All authors contributed to the drafting of the paper and approved the final version.

## Funding

This study was supported by the MRC Confidence in Concept Fund and the ISSF Wellcome Trust Grant 105603/Z/14/Z (GL-M). The MALDI Biotyper Sirius® system was provided on loan by Bruker Daltonics GmbH & Co. KG. WT is supported by The Oli Hilsdon Foundation through The Brain Tumour Charity, grant number (GN-000595).

## Conflict of interest

GL-M and LD are co-inventors of the rapid detection of colistin resistant bacteria, for which a patent has been filed by Imperial Technology Transfer (WO2018158573A1 METHOD OF DETECTION). MK is an employee of Bruker Daltonics GmbH & Co. KG, the manufacturer of the MALDI-TOF MS system used in this study, MBT Lipid Xtract™ Kit.

The remaining authors declare that the research was conducted in the absence of any commercial or financial relationships that could be construed as a potential conflict of interest.

## Publisher’s note

All claims expressed in this article are solely those of the authors and do not necessarily represent those of their affiliated organizations, or those of the publisher, the editors and the reviewers. Any product that may be evaluated in this article, or claim that may be made by its manufacturer, is not guaranteed or endorsed by the publisher.

## Footnotes

^1^ www.phytoquest.co.uk
